# Copy Number Variations and Gene Mutations Identified by Multiplex Ligation-Dependent Probe Amplification in Romanian Chronic Lymphocytic Leukemia Patients

**DOI:** 10.3390/jpm13081239

**Published:** 2023-08-09

**Authors:** Beata Balla, Florin Tripon, Marcela Candea, Claudia Banescu

**Affiliations:** 1 Department of Medical Genetics, George Emil Palade University of Medicine, Pharmacy, Science and Technology of Targu Mures, 540139 Targu Mures, Romania; beakardos@gmail.com (B.B.); claudia.banescu@gmail.com (C.B.); 2 Center for Advanced Medical and Pharmaceutical Research, Genetics Laboratory, George Emil Palade University of Medicine, Pharmacy, Science and Technology of Targu Mures, 540139 Targu Mures, Romania; 3 Department of Internal Medicine, George Emil Palade University of Medicine, Pharmacy, Science and Technology of Targu Mures, 540139 Targu Mures, Romania; marcela1212ro@yahoo.com; 4 Medical Genetics Laboratory, Emergency County Hospital of Targu Mures, 540136 Targu Mures, Romania

**Keywords:** CLL, MLPA, CNV, NOTCH1, SF3B1, MYD88

## Abstract

Chronic lymphocytic leukemia (CLL) is known for its wide-ranging clinical and genetic diversity. The study aimed to assess the associations between copy number variations (CNVs) and various biological and clinical features, as well as the survival rates of CLL patients and to evaluate the effectiveness of the multiplex ligation-dependent probe amplification (MLPA) technique in CLL patients.DNA was extracted from 110 patients, and MLPA was performed. Mutations in *NOTCH1*, *SF3B1*, and *MYD88* were also analyzed. A total of 52 patients showed at least one CNV, 26 had at least one somatic mutation, and 10 presented both, CNVs, and somatic mutations. The most commonly identified CNVs were del(114.3), del(11q22.3), and dup(12q23.2). Other CNVs identified included del(17p13.1), del(14q32.33), dup(10q23.31), and del(19p13.2). One patient was identified with concomitant trisomy 12, 13, and 19. *NOTCH1* and *SF3B1* mutations were found in 13 patients each, either alone or in combination with other mutations or CNVs, while *MYD88* mutation was identified in one patient. Forty-two patients had normal results. Associations between the investigated CNVs and gene mutations and patients’ overall survival were found. The presence of *NOTCH1* and *SF3B1* mutations or the combination of *NOTCH1* mutation and CNVs significantly influenced the survival of patients with CLL. Both mutations are frequently associated with different CNVs. Del(13q) is associated with the longest survival rate, while the shortest survival is found in patients with del(17p). Even if MLPA has constraints, it may be used as the primary routine analysis in patients with CLL.

## 1. Introduction

Chronic lymphocytic leukemia (CLL) is a form of cancer that exhibits significant clinical and genetic diversity [1,2,3].

B-cell CLL (B-CLL) consists of a clonal population of CD19+ CD5+ B cells found primarily in the peripheral blood and is one of the most common hematological disorders in adults, with an estimated incidence of approximately 4.5 new cases per 100,000 individuals annually [4]. The number of cases rises dramatically with age, exceeding 30 cases per 100,000 per year in people over 80 years old. Almost 70% of patients with CLL in the USA are over 65 years old at diagnosis, and the median age at diagnosis is 72 years. In Europe, the median age at diagnosis is 71 years in men and 74 years in women [5]. According to other sources, the average age at diagnosis is 72 years, and approximately 10% of CLL patients are under 55 years of age. The risk of developing CLL increases progressively with age, is twofold higher in men than in women, and family members of CLL patients inherit a six- to ninefold higher genetic risk [4,6,7,8].

### 1.1. Copy Number Variations

About 80% of patients with CLL usually have one of the four known chromosomal aberrations, also called copy number variations (CNVs): deletion 13q14; the deletion of region 22–23 on chromosome 11 located at the level of the long arm (11q22–23); the deletion of the 17p13 chromosomal region; and the trisomy of chromosome 12 [9]. These regions contain genes important in the pathogenesis of CLL (ex.: *RB1*, *ATM*, *TP53*, etc.). Research has shown that 50–60% of patients have 13q14 deletion, which is the most common genetic alteration occurring in CLL, and this is supported by the fact that the lost regions encode the microRNA15A and microRNA16A microRNAs, which have an inhibitory effect on the regulation of apoptosis [9,10,11]. The *RB1* gene (retinoblastoma transcriptional corepressor 1), also found on chromosome 13, functions as a cell cycle inhibitor and was the initial discovery among tumor suppressor genes. Mutations in this gene are responsible for various types of cancer in children, including retinoblastoma (RB), as well as bladder cancer and osteogenic sarcoma [12]. The deletion of 11q23 is associated with a mutation in the *ATM* (ataxiatelangiectasia mutated) gene, and is detected in 5–20% of CLL patients. The deletion can have variable sizes and develops in 30% of cases with relapse [9,13]. The protein encoded by *ATM* is believed to serve as a central regulator of the signaling pathways involved in cell cycle checkpoints. These pathways are essential for cellular responses to DNA damage and for maintaining the stability of the genome [14]. Trisomy 12 is seen in 10–20% of CLL cases and is usually associated with other chromosomal abnormalities [15,16]. The deletion of 17p in region 1, band 3 (del(17p13)) is observed in approximately 3–8% of CLL patients and is frequently associated with cases of refractory CLL, in whom the frequency of this deletion is 30% [10,15]. The role of *TP53* in cancer development is widely known and studied. It produces a tumor suppressor protein that responds to a range of cellular stresses by regulating the expression of target genes, which can lead to cell cycle arrest, apoptosis, senescence, DNA repair, or alterations in metabolism. Mutations in this gene have been linked to numerous human cancers [17].

### 1.2. Somatic Mutations

Numerous small changes (of a few nucleotides) have also been reported in the literature as possible prognostic markers. The immunoglobulin heavy chain variable region (*IGHV*) mutational status and the tumor protein p53 (*TP53*) gene have been the most important prognostic molecular markers in CLL for a long time [18,19,20,21,22]. However, new candidate genes have recently been described in CLL patients [23].

The use of next-generation sequencing (NGS) techniques in recent years has revealed the extensive genetic and epigenetic heterogeneity present in CLL [24]. The newly discovered mutations include neurogenic locus notch homolog protein 1 (*NOTCH1*), tumor protein p53 (*TP53*), splicing factor 3B subunit 1 (*SF3B1*), ataxia telangiectasia mutated (*ATM*), myeloid differentiation primary response gene 88 (*MYD88*), chromodomain-helicase-DNA-binding protein 2 (*CHD2*), and baculoviral IAP repeat-containing 3 (*BIRC3*). These recurring somatic mutations are involved in key cellular pathways such as cell cycle regulation, DNA damage response, RNA metabolism, apoptosis, nuclear factor (NF)-kB signaling, *NOTCH1* signaling, inflammatory BCR pathways, and chromatin remodeling. The *TP53*, *NOTCH1*, *SF3B1*, and *BIRC3* among these mutations have been established as prognostic factors for the course of CLL, and it is recommended to incorporate them into CLL prognostic scales [24,25]. Further recurrent mutations have been reported at frequencies below 10% in genes such as *MYD88* [26]. Mutations in genes like *FBXW7* (F-Box and WD40 domain protein 7) and *XPO1* (exportin 1) have also been described in CLL patients [21]; however, the impact of these genetic mutations remains elusive, as data on CLL patients are currently limited [26]. The corresponding proteins have different roles in the cell and are a part of different pathways: *FBXW7* in *NOTCH* signaling,* MYD88* in the immune response, *XPO1* in nucleocytoplasmic transport, etc. [21,27,28,29].

#### 1.2.1. *SF3B1* and *NOTCH1*

Besides *TP53*, the two most studied genes are *SF3B1* and *NOTCH1*. *SF3B1* mutation occurs with a frequency of 3–10% in newly diagnosed CLL patients and up to 20% in relapsed and fludarabine-refractory patients [30]. *SF3B1* encodes for subunit 1 of the splicing factor 3b protein complex. This complex, along with splicing factor 3a and a 12S RNA unit, forms the U2 small nuclear ribonucleoproteins complex (U2 snRNP). *SF3B1* mutations have been identified in several myeloid malignancies, particularly myelodysplastic syndromes (MDS), as well as other blood cancers, breast cancer, and uveal melanoma [31]. *SF3B1* mutations have been reported to impact alternative branchpoint recognition and affect various signaling pathways such as DNA damage response, telomere maintenance, NF-kB, *NOTCH1*, and MYC signaling. However, there is currently no consensus or clear understanding of the pathological mechanism(s) resulting from these mutations [32].

*NOTCH1* encodes a protein that belongs to the *NOTCH* family. These proteins are characterized by a type I transmembrane structure, which includes an extracellular domain comprising of multiple epidermal growth factor-like (EGF) repeats, and an intracellular domain made up of multiple domains [33]. The initial observation of *NOTCH1* receptor activation in malignancies was made in T-cell acute lymphoblastic leukemia with a t(7;9)(q34;q34.3), which led to its identification as a constitutively active signal. Since then, further studies have revealed that *NOTCH1* receptor activation also occurs in various solid tumors, including colorectal cancer, head and neck cancer, lung cancer, and melanoma, as well as in other hematologic malignancies such as chronic lymphocytic leukemia, mantle cell lymphoma, and Hodgkin’s lymphoma [33,34]. Clinically, patients with *NOTCH1* mutations often exhibit poorer clinical outcomes and a higher risk of disease progression. However, some studies have also reported tumor-suppressive effects of *NOTCH1*, such as its deficiency in the skin leading to the development of skin tumors. Thus, the role of *NOTCH1* signaling in cancer inhibition or promotion is multifaceted and depends on the influence of the cellular microenvironment [34]. Gaining insights into the specific strategies that CLL cells employ to exploit *NOTCH1* signaling could offer valuable hints in developing targeted treatment approaches for effectively managing CLL. *NOTCH1* mutations have been established as both a confirmed prognostic marker and a possible predictive indicator for anti-CD20-based therapies. This is because abnormalities in the Notch pathway can grant the neoplastic clone a notable growth and survival advantage [35]. During therapy, CLL patients treated with ibrutinib exhibited a gradual decrease in *NOTCH1* activity, which was later reestablished upon relapse and persisted in cases of ibrutinib-resistant disease [36]. From a treatment perspective, achieving a direct and effective therapeutic targeting of the *NOTCH1* pathway has posed challenges, and even with ibrutinib, definitive results remain elusive. However, the realm of therapeutic approaches targeting Notch signaling is rapidly evolving, offering potential novel methods such as focusing on DLL4–*NOTCH1* interactions or exploring combination therapies to effectively impact the Notch pathway in CLL [37].

#### 1.2.2. Guidelines

The International Workshop on Chronic Lymphocytic Leukemia guidelines, published in 2018, already included the assessment of *TP53* mutations in routine practice. However, evaluating other molecular targets, such as *NOTCH1*, *SF3B1*, and *BIRC3* mutations, is not yet part of the routine prognostic workup in CLL. Nevertheless, for clinical trials, molecular testing is recommended before treating a patient on a protocol [25]. The National Comprehensive Cancer Network (NCCN)’s guidelines, updated on 5 January2023, recommend testing for trisomy 12, del(11q), del(13q), del(17p), *TP53* sequencing, and molecular analysis to detect *IGHV* mutation status to assess prognosis [38].

#### 1.2.3. MLPA Analysis

CNVs are frequently found in CLL patients. Several techniques may identify those anomalies, but each of them has limitations. Multiplex ligation-dependent probe amplification (MLPA) is a multiplex PCR-based assay for the detection of CNVs in genomic DNA [39,40]. MLPA is an accurate and reliable technique for identifying CNVs, including large and small deletions, as well as single-nucleotide aberrations, with several advantages over other detection methods [41,42,43,44]. MLPA has previously proven to be a useful method in other hematological malignancies such as acute myeloid leukemia [45] and lymphoblastic leukemia [46].

The aim of this study was to investigate the associations between CNVs and various biological and clinical features, as well as the survival rates of CLL patients. We also examined the presence of somatic mutations in the *SF3B1*, *NOTCH1*, and *MYD88* genes, as well as the relation between clinical characteristics of the investigated cases and the number of genetic alterations identified. Another aim of the study was to evaluate the effectiveness of the MLPA technique in the clinical prognosis of CLL patients.

## 2. Results

**Figure 1:**
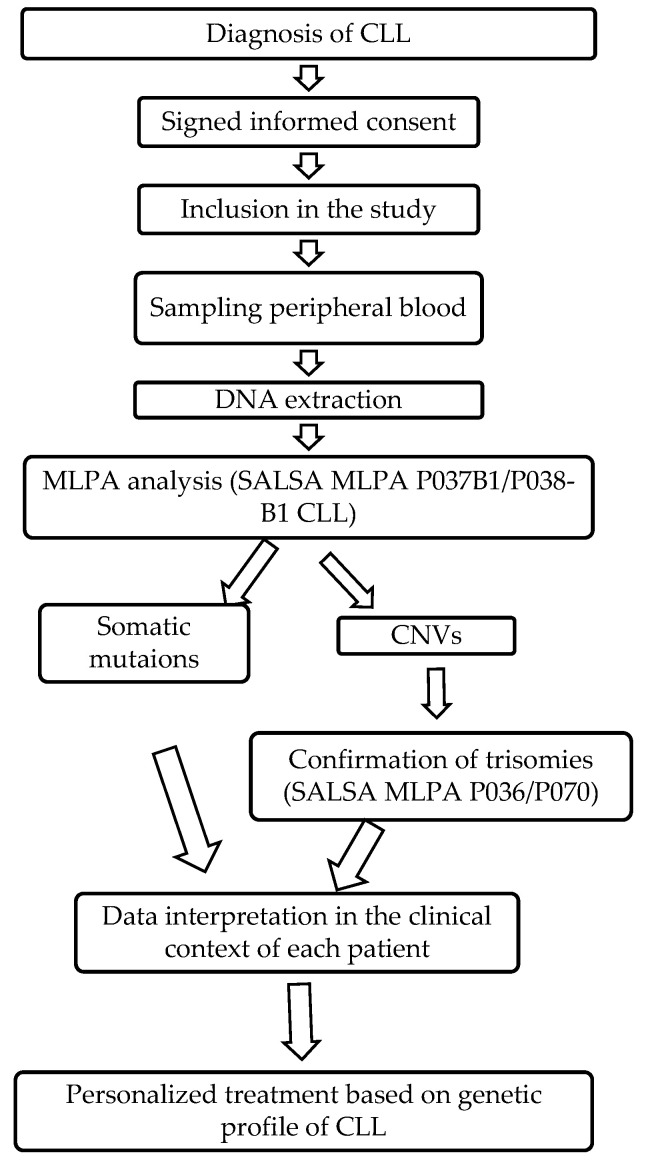
Study flowchart.

A comprehensive flowchart of the study is presented in Figure 1.

### 2.1. Clinical Characteristics of Patients

The age of the patients had a mean value (SD) of 65.5 (10.6) years (range 34–87 years). No differences in age and gender distribution were observed between patients with and without CNVs (*p* = 0.12 and *p* = 0.59, respectively). However, a statistically significant association was observed in CLL patients having somatic mutations; they appeared more frequently in patients over 60 years old (*p* = 0.04). Regarding their clinical characteristics, patients with CNVs and somatic mutations had a higher white blood count compared to those with normal genetic results (*p* = 0.03). Survival was clearly influenced by the presence of CNVs (*p* < 0.001), somatic mutations (*p* = 0.01), and their co-occurrence (*p* = 0.05). Table 1 provides a description of the age and gender distribution, along with other clinical characteristics of the patients.

In the initial stages of CLL, patients are often asymptomatic. When symptoms appear, they usually prompt clinical evolution of the disease, eventhough they are subtle and non-specific such as weakness, fatigue, loss of appetite, weight loss, fever, or night sweats. Lymphadenopathy was a prevalent feature in our study, observed in over 50% of patients, with a higher occurrence (73%) in those with associated somatic mutations, 44% in those with CNVs, and 63% in patients with both CNVs and somatic mutations (*p* < 0.05). This lymphadenopathy presented as either localized, mainly affecting cervical and supraclavicular nodes, or as generalized enlargement. Notably, we found a significant association between lymphadenopathy and the presence of *NOTCH1* mutation (*p* < 0.001), observed in more than 78% of patients. In contrast, it appeared less frequently in patients with del(13q), being present in only 36% of cases, and in those with normal MLPA results, where it affected 35% of patients. Among the other CNVs, it was noticeable that patients with del(17p) tended to have generalized lymphadenopathy. However, the prevalence did not seem to increase significantly with the association of multiple CNVs or CNVs with somatic mutations when compared to the high prevalence seen in *NOTCH1* mutations alone. Splenomegaly and hepatomegaly were less prevalent compared to lymphadenopathy in our study group, but still, a higher prevalence could be observed in those with *NOTCH1* mutation (59%), compared to those with normal results (24%) or CNVs (38%).

### 2.2. Results of MLPA Analysis

Among the 110 patients included in the study, 52 (47%) had at least one CNV, 26 (24%) had at least one somatic mutation, and 10 (9%) had both CNVs and somatic mutations. The most frequent CNVs detected were del(13q14.3) alone or in combination with other CNVs and/or somatic mutations (32 patients, 61% of those with CNVs); del(11q22.3) alone or in combination with other CNVs and/or somatic mutations (14 patients, 26%); dup(12q23.2) alone or in combination with other CNVs and/or somatic mutations (11 patients, 21%, trisomy 12 was confirmed in 8 patients); del(17p13.1) alone or in combination with other CNVs and/or somatic mutations (five patients, 9%); and del(14q32.33) alone or in combination with other CNVs and/or somatic mutations (four patients, 8%). The duplication of the 10q23.31 region was identified in two patients, in one case associated with dup(12q23.3) and in the other patient associated with del(13q14.3). One patients was found with del(11q22.3), del(17p13.1), and del(19p13.2). In addition, one patient was identified with an association of several aneuploidies, namely trisomy 12, trisomy 13, and trisomy 19.

Of the investigated somatic mutations, *NOTCH1* and *SF3B1* gene mutations were found in 13 patients each (50–50% of patients with somatic mutations), alone or in combination with other mutations or CNVs. *MYD88* gene mutation was identified in only one patient. In total, we identified genetic alterations in 68 (62%) patients, while 42 patients (38%) had no genetic alterations identified by MLPA analysis.

We also examined how the clinical characteristics and survival were affected by the number of genetic alterations identified (Table 2 and Table 3). We found that patients with more than one CNV or *SF3B1* mutation had higher white blood cell counts (*p* = 0.03 and *p* = 0.05, respectively). We also observed statistically significant correlations between genetic aberrations and patient survival in patients with *NOTCH1* and *SF3B1* mutations (*p* = 0.05) and those with a combination of *NOTCH1* mutation and CNVs (*p* = 0.02).

Furthermore, Table 4 describes the frequency of identified genetic alterations and the associations between different CNVs and somatic mutations. Here, we found that *NOTCH1* mutation was associated with CNVs in five cases (39%), especially with trisomy 12 (23%), and *SF3B1* mutation was also associated with different CNVs in five cases (39%). The co-occurrence of the two mutations was found in one patient. Del(13q) was associated with other CNVs in 13 cases (41%), mostly with del(11q) (in 28%). However, dup(10q) never occurred solely, but only in association with other CNVs and *SF3B1* mutation.

### 2.3. Survival of Patients

Overall survival of patients was 48 months, with the longest survival being observed in patients with del(13q) alone (64 months), followed by those with del(13q) associated with other CNVs (53 months), those with del(11q) (52 months), those with normal results (48 months), those with dup(12q) and trisomy 12 (36 months), those with del(14q) (11 months), and the shortest survival was noted in patients presenting with del(17p) (6 months). Among patients with somatic mutations, survival was longer in those with an *SF3B1* mutation compared to a *NOTCH1* mutation (49 versus 19 months). Short survival was also observed in patients with three CNVs (17 months) and those with a CNV and somatic mutations (31 months). Patients with a *NOTCH1* mutation associated with trisomy 12 had a mean survival of 26 months.

**Figure 2:**
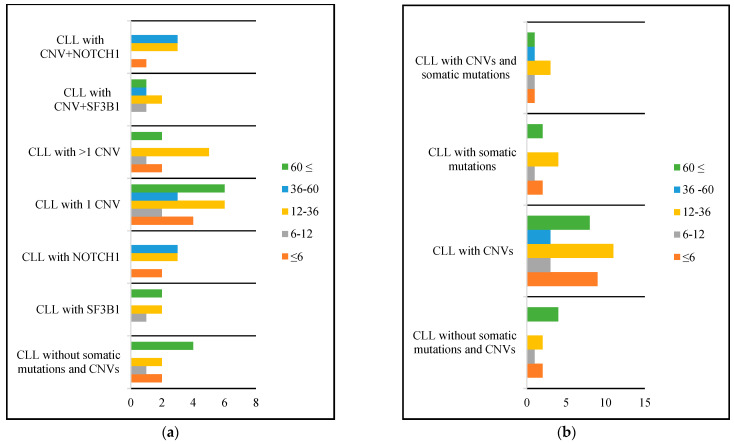
Survival of patients with CLL based on the genetic aberrations identified, presented in months. (**a**) Survival depending on the number of genetic aberrations; (**b**)survival depending on association of CNVs and somatic mutations;CLL—chronic lymphocytic leukemia; CNV—copy number variation.

Further details regarding the survival calculated only for deceased patients from our sample group are presented in Figure 2. The depicted figure illustrates the quantity of patients with survival under 6 months, between 6 and12 months, between 12 and 36 months, between 36 and 60 months, and over 60 months, categorized according to different genetic aberrations.

## 3. Discussion

### 3.1. Clinical Characteristics of CLL Patients

The mean age of CLL patients in our study was 65.5 years, which is similar to several previously published studies that describe the development of CLL in later adulthood [5,15,47,48,49]. Moreover, somatic mutations have also been found more frequently in patients over 60 years old. This fact can be explained by the results of the deep sequencing of the genome in healthy individuals, which has revealed that as humans age, one or more hematopoietic stem and progenitor cells (HSPCs) may expand and contribute more significantly to the production of mature blood cells. This phenomenon is known as age-related clonal hematopoiesis (ARCH) and is characterized by the expansion of HSPC clones with specific genetic mutations that can increase the risk of certain diseases, such as leukemia, heart disease, and diabetes [50]. Recent research has shown that ARCH may also be associated with the expansion of B-cell clones with CLL phenotype. However, larger-scalestudies are needed to confirm this association and gain a deeper understanding of the pathogenesis of these conditions [50]. Further recent studies have shown that as individuals age, their tissues experience an increase in somatic mutations, which can lead to clonal hematopoiesis if it occurs in the hematopoietic system [51]. Chronic lymphocytic leukemia (CLL) and losses of sex chromosomes are common variants that accumulate in aging. However, the mechanisms behind why these variants are positively selected during aging remain unclear, particularly in cases where clonal hematopoiesis occurs without a known driver mutation. This could be due to mutations in unexplored genes or the non-coding genome, or a genetic drift resulting from age-related restriction of the stem cell pool [51].

Regarding further clinical characteristics of CLL patients, we found that a higher white blood cell count was associated with the presence of CNVs and somatic mutations, and therefore with a lower survival rate. These results are sustained by other studies which describe an elevated white blood cell count as an unfavorable prognostic factor [52].

### 3.2. CNVs in CLL Patients

Our MLPA results showed that the most frequent genetic aberration in CLL patients was del(13q14.3), followed by del(11q22.3), dup(12q23.2) (trisomy 12 confirmed in most cases), and del(17p13.1). They are similar to those previously published, although there are slight differences in the order of frequency of the four most common genetic aberrations. Eid et al. found that the deletion of 13q14 was followed by trisomy 12, the deletion of 11q22,3, and the deletion of 17p13 (*TP53*) [53], while in other studies, the most common aberration was also the 13q14.3 deletion (44%), whereas 11q and 17p deletions and trisomy 12 were less frequent (5%, 2%, and 7%, respectively) [54,55]. Our results are in accordance with the abovementioned studies regarding the survival of CLL patients as well, since the most favorable prognosis was seen in patients with del(13q), followed by del(11q) and trisomy 12. Additionally, we identified del(14q32.33) that has been rarely described in CLL patients [56,57]. The Mitelman Database for Chromosome Aberrations and Gene Fusions in Cancer (available at: https://mitelmandatabase.isb-cgc.org/ accessed on 1 June 2023) to this date has 120 CLL cases registered with the deletion of 14q, besides other balanced structural aberrations involving this region. In our study, we were able to identify this CNV in four patients in two cases associated with del(13q), in one case associated with del(17p), and in one case as a sole aberration.

According to our knowledge, this is the first study describing the presence of dup(10q23.31) in CLL patients, in one case associated with del(13q), and in the other case associated with dup(12q). The two probes of the MLPA probemix used for this chromosomal region are specific for the *PTEN* gene. *PTEN* is a versatile tumor suppressor that is frequently absent in human cancer, with varying degrees of occurrence in prostate cancer, glioblastoma, endometrial, lung, and breast cancer. The gene’s encoded protein acts as a tumor suppressor by inhibiting the AKT/PKB signaling pathway [58]. The deletion or inactivation of *PTEN* has been previously described in CLL patients as well [59,60]. A study by Nicolas L. et al. states that B-CLL frequently experiences the loss of heterozygosity or allelic imbalances at 10q23.3, but *PTEN* gene is not affected by it. However, the absence of PTEN protein may still occur in B-CLL despite having a normal *PTEN* genotype. This implies that the phosphatase has a potential role in the molecular pathology of B-CLL [61]. The duplication of the 10q region has been described in very few acute myeloid leukemia (AML) patients [62,63]. Given the abovementioned, this unusual finding in CLL patients needs further studies for a better understanding of the underlying mechanisms.

We identified one case of trisomy 19, associated with trisomy 12 and 13. The association of trisomy 19 andother trisomies with trisomy 12 has been previously described by other authors [64,65,66]. Furthermore, the Mitelman Database for Chromosome Aberrations and Gene Fusions in Cancer has multiple studies registered where the association of trisomy 12, 13, and 19 along with other trisomies is present.

The lowest survival rate in our study was found in patients with del(17p), a region that contains the *TP53* gene. It is well known that abnormalities in *TP53* are among the most significant prognostic and predictive markers used to assess prognosis and guide treatment decisions in CLL. These abnormalities are linked to significantly reduced survival rates and a diminished response to chemoimmunotherapy [3,67,68,69]. Therefore, our results support the results of numerous previously published studies on this topic.

### 3.3. Somatic Mutations in CLL Patients

Data suggest that *SF3B1* mutations are associated with a more aggressive disease progression and a shorter survival in CLL, independent of other prognostic factors. One indication of the potential impact of *SF3B1* mutation on CLL comes from the observation that *SF3B1* mutation significantly co-occurs with del(11q) [70,71]. Our results, in accordance with these theories, showed a relatively frequent association with CNVs (five out of 13; 39%), mainly with del(11q) and del(13q) (23–23% of patients with *SF3B1* mutation, for each CNV). Patients with an association of *SF3B1* mutation and del(11q) showeda mean survival of 43.66 months, compared to 71 months in those with only *SF3B1* mutation.

Mutations in *NOTCH1* have been described in 5–10% of newly diagnosed CLL patients, with increasing frequency in advanced disease stages. Patients with *NOTCH1* mutations have a shorter survival independent of other prognostic factors and are often associated with trisomy 12 (41.9%) [72]. The mutation investigated in this study is a2-bp frameshift deletion in exon 34 (c7541_7542delCT) that generates a premature stop codon (P2514fs^*^4), thus truncating the PEST (proline–glutamic acid–serine–threonine-rich) domain. This mutation has been previously linked to NOTCH signaling activation in other studies [73,74]. *NOTCH1*-mutated patients have previously been associated with poor response to chemoimmunotherapy and aggressive disease biology in studies [24,75]. Our results are in line with these studies, as our patients with *NOTCH1* mutations showed lower survival rates compared to those with normal results.

### 3.4. Co-Occurrence of CNVs and Somatic Mutations in CLL Patients

Multiple genetic aberrations have been well documented in CLL, contributing to disease heterogeneity [76]. In our study, we identified three cases with a co-occurrence of del(11q) and *SF3B1* mutation, and the median survival of these cases was 44 months, which was lower than that of patients with *SF3B1* mutation or del(11q) alone. This may sustain the idea that the co-occurrence of a CNV and somatic mutation is associated with a poorer prognosis. Furthermore, our results indicate that such co-occurrences confer a poorer prognosis than any CNVs alone, except for del(17p).

Trisomy 12 is CLL’s third most frequent chromosomal aberration and confers an intermediate prognosis. In a cohort of 104 untreated patients carrying trisomy 12, *NOTCH1* mutations occurred in 24% of cases and were associated with unmutated *IGHV* genes (*p* = 0.003) and trisomy 12 as a sole cytogenetic abnormality (*p* = 0.008). The co-occurrence of *NOTCH1* mutations with trisomy 12 was associated with an approximately 2.4-fold increase in the risk of death, a significant shortening of survival (*p* < 0.01), and proved to be an independent predictor of survival in multivariate analysis [77]. Trisomy 12 associated with *NOTCH1* mutations has been shown to potentially explain the poorer prognosis of trisomy 12 cases compared to del(13q) cases [66,72,77]. Our results similarly identified three cases of *NOTCH1* mutation associated with trisomy 12. Given the fact that some of our *NOTCH1*-mutated patients have been diagnosed recently and are alive, we cannot compare survival with those of other studies. Survival rate needs a re-evaluation when a longer follow-up is available.

### 3.5. Techniques for CNV Investigation: Benefits, Challenges, and Limitations

In recent years, MLPA has been successfully applied as a tool in cancer research, providing precise information on increased or decreased copy numbers at specific loci [39,78]. It can provide detailed multiplex profiles of chromosomal aberrations in tumor samples in a relatively short period of time [64]. MLPA allows the detection of targeted CNVs in multiple human genes concurrently, and it has been used to screen CNVs in previous studies [54,79]. CNVs are regarded as a major source of structural variation in the genome and range in size from 50 base pairs (bp) to several megabases(Mbs) [43,44]. CNVs are estimated to contribute to 4.8–9.5% of the genome and can affect gene expression levels or induce chromosomal rearrangements causing various disorders and diseases [80,81,82].

The MLPA probemix used in this study contains probes targeting all genomic regions recommended to be tested by the most recent NCCN guidelines; in addition, it targets further genomic areas known to be linked to CLL; therefore, it is a more comprehensive testing tool in evaluating CLL patients.

#### 3.5.1. MLPA and Conventional Cytogenetics

Nardinelli et al. conducted a study comparing MLPA and conventional cytogenetics and concluded that MLPA identified more abnormalities than cytogenetics (44%). Cytogenetic analysis revealed 25 aberrant karyotypes, whereas 41 karyotypes were normal. MLPA analysis detected genetic abnormalities in 45 cases, and 22 had normal results. Their findings highlight the advantage of MLPA compared with conventional cytogenetics. In conclusion, they state that MLPA is a reliable high-throughput technique to detect CNVs and is also one that is cost-effective, able to be included in routine diagnostic protocols in laboratories that lack conventional cytogenetics [83].

#### 3.5.2. MLPA and FISH

Another study conducted by Ola M et al. in 2021 compared MLPA and FISH (fluorescencein situ hybridization) techniques in evaluating 30 CLL patients. Their results showed that in 80% of cases, the MLPA and FISH results were concordant. In 70% of the cases, FISH detected aberrations while no abnormalities were found in 30% of the cases. On the other hand, MLPA detected aberrations in 66.7% of the cases, and no abnormalities were found in the remaining 33.3% of cases. Discordant results were attributed to a low percentage of mosaicism, and they concluded that the MLPA technique is disadvantaged by its inability to detect targeted abnormalities in a sample with a low level of mosaicism. However, the restricted number of regions that can be evaluated by FISH is considered a disadvantage. MLPA is more cost-efficient than FISH and encompasses a broader range of target gene loci. Therefore, they recommend the usage of MLPA as the first screening platform [53].

Our study validates the aforementioned conclusions, as our MLPA analysis demonstrated a high detection rate of genetic aberrations in 62% of patients across 51 different loci. Nevertheless, the detection rate of 62% compared to the high frequency of anomalies described in CLL may be due to the presence of low-level mosaic aberrations (<25–30% of cells with anomalies/aberrant cells).

#### 3.5.3. FISH and aCGH

High-resolution genome-wide detection of CNVs can also be achieved through the use of genomic arrays, making them an effective tool for identifying complex karyotypes [84]. Array-based comparative genomic hybridization (aCGH) and single nucleotide polymorphism (SNP) arrays are genomic array platforms that can be used to screen the entire genome for CNVs in a single experiment. These platforms can identify CNVs ranging from 10 to 100 kb and have been utilized in the diagnostic evaluation of hematologic malignancies, such as CLL. In a study published by Alexander C.L. et al., the authors compared CGH with fluorescence in situ hybridization (FISH) detection and chromosome banding analysis (CBA) in CLL, and they found that in 34% of patients, CNVs were identified outside of the regions captured by FISH probes, and some of these alterations, such as gains in 8q and deletions of 9p and 18p (*p* < 0.01), were associated with a negative prognosis. In a subset of 260 patients, where both FISH and genomic array results were available from the same sample, a concordance rate of 92.2% between the two methods was described [85]. Alexander C.L. et al. concluded that genomic arrays were shown to identify more chromosomal abnormalities and performed equally well as simultaneous chromosome banding analysis in terms of risk stratification. Conducting a genome-wide analysis of genetic aberrations also offers valuable supplementary prognostic details when compared to FISH analysis alone, thereby enhancing the risk-adapted stratification of patients [85]. Therefore, CGH analysis may be effective for identifying multiple CNVs; however, it lacks the possibility to analyze the presence of somatic mutations.

#### 3.5.4. MLPA and aCGH

In a study published by D. Adão, MLPA-targeted probes were used to validate the aCGH results. By this study, they confirmed that all CLL characteristic alterations identified by aCGH were also confirmed by MLPA. This demonstrates that both techniques can be used for the further characterization of samples. However, aCGH is more expensive and time-consuming than MLPA, making the latter a more advantageous option. Additionally, MLPA has the ability to detect eight CLL alterations beyond the four most common ones that are assessed by FISH, which include 2p gain, del(6q), 8p loss, 8q gain, del(9p21), *PTEN* deletion at 10q23.31, del(14q), and trisomy 19. Moreover, MLPA can also identify mutations on *NOTCH1*, *SF3B1*, and *MYD88* [57].

Our study has some limitations, including a small sample size and a lack of information regarding translocations, which are rare events in CLL. Additionally, our study lacks data regarding *IGHV* mutations or sequence changes (SNPs) that may remain undetected using MLPA-based methods.

Although our study has limitations, we have several strengths, such as employing a combination of suitable SALSA MLPA kits that enabled us to identify a significant proportion of anomalies in our sample group. These included deletions, duplications, aneuploidy, and point mutations.

## 4. Materials and Methods

The study included 110 CLL patients from the central region of Romania. Peripheral bloodsamples were collected at diagnosis when the patients were admitted to the hematology departments in Târgu Mureș, before the start of chemotherapy (considered treatment-naive). Inclusion criteria were as follows: diagnosis of CLL, age older than 18 years, and signed informed consent to participate in the study.

To extract genomic DNA, we used Pure Link Genomic DNA kits from Thermo Fischer Scientific (Waltham, MA, United States), and followed the manufacturer’s instructions. The MLPA technique, which is a multiplex polymerase chain reaction (PCR) method, was used to identify the most prevalent CNVs linked to hematological malignancies.

The MLPA analysis was conducted following the instructions provided by the manufacturer (MRCHolland, Amsterdam, The Netherlands) using the SALSA MLPA probemix P037-B1/P038-B1 CLL, which simultaneously analyzes the following gene regions: 2p (*MYCN*, *ALK*, *REL*), 6q, 8p (*TNFRSF10A/B*), 8q (*EIF3H*, *MYC*), 9p21 (*CDKN2A/B*), 10q (*PTEN*), 11q (*ATM*, *RDX*, *PPP2R1B*, *CADM1*), chromosome 12, 13q14 (*RB1*, *DLEU1/2/7*, *KCNRG*, *MIR15A*), 14q, 17p (*TP53*), and chromosome 19. We also investigated the presence of somatic mutations in the *SF3B1*, *NOTCH1*, and *MYD88* genes, specifically the K700E mutation in *SF3B1*, the 7541-7542delCT mutation in *NOTCH1*, and the L265P mutation in *MYD88*. To confirm the presence of trisomies (an abnormality characterized by the presence of three copies of a particular chromosome), we utilized kits for the subtelomeric regions (SALSA MLPA Probemix P036 Subetlomeres Mix 1 and Probemix P070 Subtelomeres Mix B2) and centromeric regions (SALSA MLPA Probemix P181 Centromere mix 1). When using kits for the subtelomeric regions, the presence of trisomy was considered only when two probes for the short (p) and long (q) arms of the same chromosome were simultaneously gained. The presence of K700E mutation in *SF3B1* and the L265P mutation in *MYD88* were confirmed by real-time PCR, using TaqMan^®^SNP Genotyping Assays (C_365451600_10 and C_175984374_10, respectively), while the 7541-7542delCT mutation in *NOTCH1* was validated by amplification refractory mutation system–PCR (ARMS-PCR) method (as described by Rossi et al.) [86]. Treatment given to the patients was according to the ESMO (European Society for Medical Oncology) guidelines as front-line therapy [6], the treatment strategy being adapted according to the patient and genetic risk factors. For patients with del(17p) currently in Romania, signaling pathway inhibitors such as Ibrutinib, or Idelalisib+Rituximab, and Venetoclax are used both as first-line treatments and in cases of relapse. Additionally, in “Fit” patients, the response can be strengthened through allotransplant of stem cells, if they are eligible.

The Coffalyser software from MRC Holland was used to interpret the results obtained with the SALSA MLPA kits. We used a genetic analyzer (Genetic Analyzer 3500 from Applied Biosystems, Waltham, MA, USA) to perform the MLPA technique. We used chi-squared tests to compare demographic and clinical characteristics. Statistical tests were conducted using a two-sided approach and *p* values less than 0.05 were considered statistically significant. Statistical analyses were performed using IBM-SPSS Statistics v 28.0.1.1(15). Chi-squared tests were used to calculate differences between categorical variables.

## 5. Conclusions

Our results demonstrated that elderly patients are more prone to developing the investigated somatic mutations, which can be due to age-related clonal hematopoiesis. CLL patients with CNVs and/or somatic mutation have a higher white blood cell count, which is considered an unfavorable prognostic factor.

Genetic aberrations such as somatic mutations (specifically *NOTCH1* mutation) and/or CNVs have a serious influence on the early appearance of clinical symptoms of CLL patients, and therefore might trigger the clinical evolution of the disease.

The presence of *NOTCH1* and *SF3B1* mutations or the combination of *NOTCH1* mutation and CNVs significantly influence the survival of patients with CLL, with *NOTCH1* mutation having a greater influence over survival than SF3B1 mutation. Both mutations are frequently associated with different CNVs.

Del(13q) is associated with the longest survival rate, followed by del(11q) and trisomy 12, while the shortest survival is found in patients with del(17p).

The MLPA analysis has been demonstrated as an extensive molecular cytogenetic method for identifying cytogenetic indicators of chronic lymphocytic leukemia. As a result, even if the method has constraints, it may be used as the primary routine analysis in patients with CLL.

## Figures and Tables

**Table 1:** Clinical characteristics of CLL patients.

Variable	All CLL (*n* = 110)	CLL without Somatic Mutations and CNVs (*n* = 42)	CLL with CNVs (*n* = 52)	*p* Value	CLL with Somatic Mutation (*n* = 26)	*p* Value	CLL with CNVs and Somatic Mutation (*n* = 10)	*p* Value
Age, y								
30–39	1 (1)	1 (2)	0 (0)	0.12	0 (0)	**0.04**	0 (0)	0.20
40–49	7 (6)	4 (10)	3 (6)		0 (0)		0 (0)	
50–59	28 (26)	13 (31)	14 (27)		2 (8)		1 (10)	
60–69	34 (31)	14 (33)	10 (19)		13 (50)		3 (30)	
≥70	40 (36)	10 (24)	25 (48)		11 (42)		6 (60)	
Sex								
Men	70 (64)	26 (62)	35 (67)	0.59	10 (39)	0.06	7 (70)	0.63
Women	40 (36)	16 (38)	17 (33)		16 (62)		3 (30)	
WBC, cells/µL								
<9000	14 (13)	10 (24)	4 (8)	**0.03**	1 (4)	**0.03**	1 (10)	0.34
≥9000	96 (87)	32 (76)	48 (92)		25 (96)		9 (90)	
PLT, cells/µL								
<150,000	42 (38)	14 (33)	23 (44)	0.28	8 (31)	0.83	3 (30)	0.84
≥150,000	68 (62)	28 (67)	29 (56)		18 (69)		7 (70)	
Hemoglobin, g/dL								
<13	74 (67)	31 (74)	33 (64)	0.28	18 (69)	0.68	8 (80)	0.68
≥13	36 (33)	11 (26)	19 (37)		8 (31)		2 (20)	
LDH, IU/L								
<480	90 (82)	36 (86)	40 (77)	0.28	23 (89)	0.75	9 (90)	0.72
≥480	20 (18)	6 (14)	12 (23)		3 (12)		1 (10)	
LYMPH, %								
<25	5 (5)	3 (7)	1 (2)	0.20	1 (4)	0.31	0 (0)	0.45
25–40 *	4 (4)	3 (7)	1 (2)		0 (0.)		0 (0)	
≥40	101 (92)	36 (86)	50 (96)		25 (96)		10 (100)	
Alive	43 (39)	23 (55)	4 (8)	**<0.001**	6 (23)	**0.01**	2 (20)	**0.05**
Deceased	67 (61)	19 (45)	48 (92)		20 (77)		8 (80)	

Data are presented as numbers (percentage), *p* values were obtained by the χ^2^ test. * Normal values of the laboratory.

**Table 2:** Detailed description of clinical characteristics based on the number of CNVs identified.

Variable	CLL without CNVs	1 CNV	*p* Value	>1CNV	*p* Value
110 (100)	42 (38)	35 (32)		17 (16)	
Age, y					
30–39	1 (2)	0 (0)	0.12	0 (0)	0.46
40–49	4 (10)	2 (6)		1 (6)	
50–59	13 (31)	11 (31)		3 (18)	
60–69	14 (33)	5 (14)		5 (29)	
≥70	10 (24)	17 (49)		8 (47)	
Sex					
Men	26 (62)	21 (60)	0.86	14 (82)	0.13
Women	16 (38)	14 (40)		3 (18)	
WBC, cells/µL					
<9000	10 (24)	4 (11)	0.16	0 (0)	**0.03**
≥9000	32 (76)	31 (89)		17 (100)	
PLT, cells/µL					
<150,000	14 (33)	14 (40)	0.54	9 (53)	0.16
≥150,000	28 (67)	21 (60)		8 (47)	
Hemoglobin, g/dL					
<13	31 (74)	22 (63)	0.30	11 (65)	0.48
≥13	11 (26)	13 (37)		6 (35)	
LDH, IU/L					
<480	36 (86)	27 (77)	0.33	13 (77)	0.39
≥480	6 (14)	8 (23)		4 (24)	
LYMPH, %					
<25	3 (7)	1 (3)	0.47	0 (0)	0.26
25–40 *	3 (7)	1 (3)		0 (0)	
≥40	36 (86)	33 (94)		17 (100)	
Alive	23 (55)	23 (66)	0.33	5 (29)	0.08
Deceased	19 (45)	12 (34)		12 (71)	

Data are presented as numbers (percentage), *p* values were obtained by the χ^2^ test. * Normal values of the laboratory.

**Table 3:** Detailed description of clinical characteristics based on somatic mutations identified, alone or in combination with CNVs.

Variable	CLL without Somatic Mutations	*SF3B1*	*p* Value	*NOTCH1*	*p* Value	*SF3B1* + CNV	*p* Value	*NOTCH1* + CNV	*p* Value
110 (100)	42 (38)	13 (12)		13 (12)		5 (5)		5 (5)	
Age, y									
30–39	1 (2)	0 (0)	0.15	0 (0)	0.16	0 (0)	0.86	0 (0)	0.13
40–49	4 (10)	0 (0)		0 (0)		0 (0)		0 (0)	
50–59	13 (31)	1 (8)		1 (8)		1 (20)		0 (0)	
60–69	14 (33)	9 (69)		5 (39)		2 (40)		1 (20)	
≥70	10 (24)	3 (23)		7 (54)		2 (40)		4 (80)	
Sex									
Men	26 (62)	9 (69)	0.63	7 (54)	0.60	4 (80)	0.43	3 (60)	0.93
Women	16 (38)	4 (31)		6 (46)		1 (20)		2 (40)	
WBC, cells/µL									
<9000	10 (24)	0 (0)	**0.05**	1 (8)	0.20	0 (0)	0.22	1 (20)	0.85
≥9000	32 (76)	13 (100)		12 (92)		5 (100)		4 (80)	
PLT, cells/µL									
<150,000	14 (33)	4 (31)	0.86	1 (8)	0.07	3 (60)	0.24	0 (0)	0.12
≥150,000	28 (67)	9 (69)		12 (92)		2 (40)		5 (100)	
Hemoglobin, g/dL									
<13	31 (74)	10 (77)	0.82	9 (69)	0.75	4 (80)	0.76	4 (80)	0.76
≥13	11 (26)	3 (23)		4 (31)		1 (20)		1 (20)	
LDH, IU/L									
<480	36 (86)	13 (100)	0.15	10 (77)	0.45	5 (100)	0.37	4 (80)	0.73
≥480	6 (14)	0 (0)		3 (23)		0 (0)		1 (20)	
LYMPH, %									
<25	3 (7)	1 (8)	0.61	0 (0)	0.35	0 (0)	0.66	0 (0)	0.66
25–40 *	3 (7)	0 (0)		0 (0)		0 (0)		0 (0)	
≥40	36 (86)	12 (92)		13 (100)		5 (100)		5 (100)	
Alive	23 (55)	3 (23)	**0.05**	3 (23)	**0.05**	2 (40)	0.53	0 (00)	**0.02**
Deceased	19 (45)	10 (77)		10 (77)		3 (60)		5 (100)	

Data are presented as numbers (percentage), *p* values were obtained by the χ^2^ test. * Normal values of the laboratory.

**Table 4:** Description of the genetic alterations identified in patients with CLL by MLPA.

Genetic Alteration Identified		No. Patients (%)
Somatic mutations	*NOTCH1* p.P2514*fs	12 (11)
	*SF3B1* p.K700E	12 (11)
	*MYD 88* p.L265P	1 (1)
	*NOTCH1*, *SF3B1*	1 (1)
CNVs	del(13q)	19 (17)
	del(11q), del(13q)	9 (8)
	dup(12q)	9 (8)
	del(11q)	4 (4)
	del(13q), del(14q)	2 (2)
	del(17p)	2 (2)
	del(14q), del(17p)	1(1)
	del(13q), del(17p)	1 (1)
	del(11q), del(17p), del(19p)	1(1)
	dup(10q), del(13q)	1(1)
	dup(10q), dup(12q)	1(1)
	del(14q)	1 (1)
	trisomy 12, trisomy 13, trisomy 19	1(1)
Somatic mutations and CNVs associated	*NOTCH1*, trisomy 12	3 (3)
	*NOTCH1*, del(13q)	2 (2)
	*SF3B1*, del(13q), del(11q)	2 (2)
	*SF3B1*, del(13q)	1 (1)
	*SF3B1*, dup(10q), dup(12q) *SF3B1*, del(11q)	1 (1) 1 (1)

## Data Availability

Additional data are available upon request.
